# Barrel cortical neurons and astrocytes coordinately respond to an increased whisker stimulus frequency

**DOI:** 10.1186/1756-6606-5-12

**Published:** 2012-04-26

**Authors:** Jun Zhao, Dangui Wang, Jin-Hui Wang

**Affiliations:** 1 Institute of Biophysics, Chinese Academy of Sciences, Beijing 100101, China; 2 Graduate School of the Chinese Academy of Sciences, Beijing 100049, China; 3 College of Life Science, University of Science and Technology of China, Hefei, Anhui 230026, China

**Keywords:** Neuron, Astrocyte, Barrel cortex, Whisker and two-photon cellular imaging

## Abstract

**Background:**

Nerve cells program the brain codes to manage well-organized cognitions and behaviors. It remains unclear how a population of neurons and astrocytes work coordinately to encode their spatial and temporal activity patterns in response to frequency and intensity signals from sensory inputs.

**Results:**

With two-photon imaging and electrophysiology to record cellular functions in the barrel cortex *in vivo*, we analyzed the activity patterns of neurons and astrocytes in response to whisker stimuli with increasing frequency, an environmental stimulus pattern that rodents experience in the accelerated motion. Compared to the resting state, whisker stimulation caused barrel neurons and astrocytes to be activated more synchronously. An increased stimulus frequency up-regulated the activity strength of neurons and astrocytes as well as coordinated their interaction. The coordination among the barrel neurons and astrocytes was fulfilled by increasing their functional connections.

**Conclusions:**

Our study reveals that the nerve cells in the barrel cortex encode frequency messages in whisker tactile inputs through setting their activity coordination.

## Background

Brain functions are fulfilled by the activities of nerve cells [1-3]. The emerging evidences demonstrate that the neurons and astrocytes are involved in the information processing in the central nervous system [4-8]. Their communications are critical for the brain functions, such as the astrocytes are able to regulate the neuronal activities and plasticity [9-12]. However, how these neurons and astrocytes in local networks communicate coordinately to program the brain codes for well-organized cognition and behaviors remains unclear. The address of this issue helps to reveal the principles of information processing in the brain.

The studies by two-photon cellular imaging show that sensory stimuli, such as vision and auditory, activate the groups of neurons and astrocytes in sensory cortices [6,8,13-16]. Their temporal and spatial activity patterns in response to a single stimulation are well known [17-21]. In fact, the sensory inputs include the messages of frequency and intensity [22-25]. How the neurons and astrocytes in cortical networks coordinately work to recognize such messages in sensory inputs remains elucidated.

The nerve cells in the barrel cortex receive whisker tactile inputs [26-28], which is well used to study how the nerve cells in barrel cortical networks encode the whisker inputs. With the approaches of two-photon cellular imaging and electrophysiology *in vivo*, we examined the temporal and spatial activity patterns of barrel neurons and astrocytes in response to an increasing stimulus frequency to the whiskers, a common external stimulation when rodents explore their environments, such as the accelerated motion [23,25]. By analyzing the magnitudes and temporal correlation of responses among the neurons and astrocytes, we found that neurons and astrocytes in barrel cortex coordinately respond to an increased whisker stimulus frequency.

## Results

**Figure 1:**
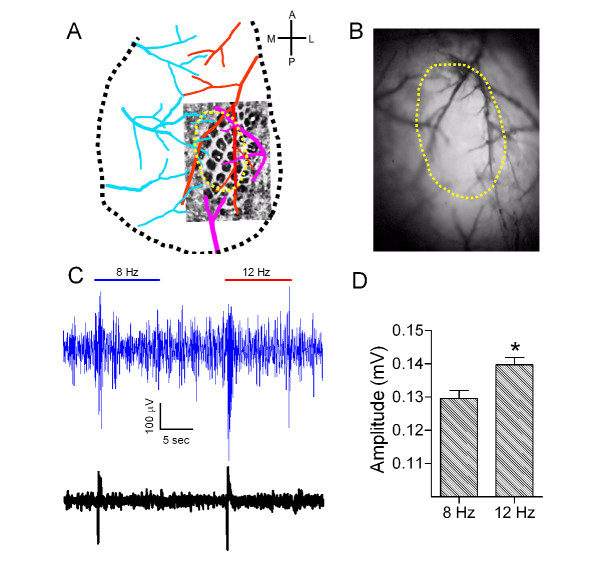
**The activity of barrel neurons rises in response to an increased frequency of whisker tactile input. A**) shows a spatial relationship between superfical vessels and barrel area in cerebral cortex. The veins are labeled in blue, the middle cerebral artery (mca) is labeled in red, and the others are pink. The location of barrel cortex is comfirmed by using histological reconstructions. **B)** shows an example of small craniotomy above the barrel cortex after removing a piece of skull. The cycles marked by yellow dash-line in A and B present the same area. **C)** shows an exmaple of LFPs induced by 8-to-12 Hz stimuli (top) and the averaged trace of 20 LFP sweeps (bottom). **D)** shows LFP amplitiutdes at 12 Hz stimuli are significantly larger than those at 8 Hz stimili (p < 0.05, students’ test,).

Local field potentials were recorded in the barrel cortices of mice to reveal how sensory neurons recognize frequency messages in whisker tactile input. We subsequently studied how the neurons and astrocytes coordinately respond to the frequency messages by analyzing their activity strength and synchrony with two-photon calcium imaging *in vivo*. The barrel cortices for the field potential recording and two-photon cellular imaging were localized as illustrated in Figure 1A ~ B. The levels of Ca^2+^ signal in the cells were presumably proportional to their activities [19,29-32]. Sulforhodanmin-101 (SR-101) that labeled the astrocytes [33] was used to isolate astrocytic signal from neuronal one. The frequency messages in whisker tactile inputs were to stimulate mouse whiskers by paired burst-stimuli with increasing frequency (stimulus one at 8 Hz and stimulus two at 12 Hz, i.e., 8-to-12 Hz). The spatial patterns of cellular activity were analyzed by the portions of Ca^2+^ signal patterns, and the synchrony was estimated by pair-wise cross-correlation.

### The activity of barrel neurons rises in response to an increased frequency of whisker tactile input

By recording local field potentials (LFP) in the barrel cortex, we studied the responses of neurons to whisker stimuli in frequencies 8-to-12 Hz. Figure 1C illustrates a sweep of LFP signals (top trace) and the averaged LFP signals (bottom trace) induced by 8-to-12 Hz stimuli. The paired responses appear an increment pattern, i.e., response two is larger than response one. In statistical analysis, LFP amplitudes are significantly larger in response to 12 Hz stimuli than to 8 Hz ones (*p* < 0.05; Figure 1D).

As an increasing frequency of stimulus-to-whisker makes neuronal responses enhanced, the network neurons discriminate the increases of input frequency through strengthening their responsiveness. How the neurons and astrocytes in the barrel cortex coordinately respond to the frequency messages was analyzed by two-photon calcium imaging *in vivo*.

### Neurons and astrocytes in barrel networks are coordinately activated by whisker tactile inputs

**Figure 2:**
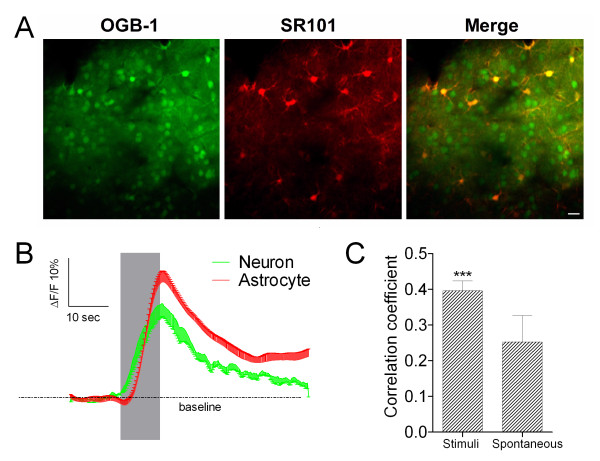
**Whisker tactile inputs induce intracellular Ca**^**2+ **^**changes in the neurons and astrocytes of mouse barre cortex. A**) shows two-photon images of these nerve cells (200 μm in the depth of barrel cortex), in which OGB-1 labels nerve cells (left panel), SR101 labels astrocytes (middle), and astrocytes are yellow in a merged image (right). Scale bar is 20 μm. **B**) shows the relative changes of OGB-1 fluorescence signals (ΔF/F) averaged from the cell bodies of neurons (green line) and astroctyes (red) in a barrel network (gray bars are the durations of whisker stimuli). **C**) illustrates a comparison of neuron-astrocyte pairswise synchrony under the conditions with (n = 8) and without stimuli (n = 6). The synchrony of stimulus-driven activities is significantly higher than that of spontaneous ones (*p* < 0.01). The synchrony of cellular activities is quantified by using the peak cross-correlation coefficient.

After OGB-1 AM was loaded into neurons and astrocytes in the barrel cortex (Methods), cellular Ca^2+^ signals were measured by two-photon laser scanning microscopy. Left, middle and right panels in Figure 2A illustrate OBG-labeled nerve cells (green), SR101-labeled astrocytes (red) and their merged imaging (yellow for the astrocytes and greens for the neurons), respectively. Figure 2B shows the stimulus-induced changes of Ca^2+^ signals (ΔF/F) averaged from the neurons (green trace) and astrocytes (red), i.e., both of them respond to whisker tactile inputs. Pairswise cross-correlations between neurons and astrocytes in their activities (Methods) were analyzed under the conditions of whisker stimuli (n = 8 mice) and no stimuli (n = 6). Correlation coefficients with vs. without whisker stimuli are 0.40 ± 0.03 and 0.25 ± 0.07, respectively (*p* < 0.01 in Figure 2C), indicating that neurons and astrocytes in the barrel cortex are coordinately activated by whisker stimuli.

We further analyzed how neurons and astrocytes in the barrel cortex coordinately respond to frequency messages in whisker tactile inputs by measuring their spatial and temporal activities. This analysis is based on the data that the astrocytes in the brain regulate neuronal excitability and synaptic functions [34-36].

### Barrel neurons and astrocytes increase their activity strength in response to high frequency input

In the study of the coordinate responses of neurons and astrocytes to frequency messages in whisker tactile inputs, we compared the changes of their activity strength induced by pair burst-stimuli to whiskers in frequencies 8-to-12 Hz (each burst for 10 sec. and inter-burst intervals in 10 sec.). Response one (R1) was corresponding to 8 Hz stimulus, and response two (R2) was a response to 12 Hz. Compared to control (8-to-8 Hz) in Additional file 1: Figure S1 ~  Additional file 2: Figure S 2, an increased stimulus frequency appears to enhance the responsive strength in the neurons and astrocytes.

**Figure 3:**
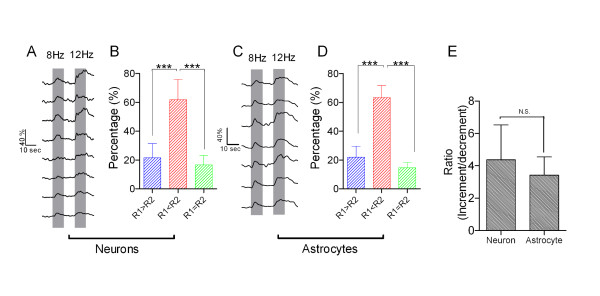
**Barrel network neurons and astrocytes show a dominant increment in responses to the frequency increase of paired whisker stimuli. A**) shows two sequential Ca^2+^ signals at the barrel neurons (a trace per cell) in response to paired burst-stimili at 8-to-12 Hz. **B)** The percentages of the three patterns among barrel network neurons (n = 5 mice) in response to 8-to-12 Hz stimuli show the increment in dominance. **C)** Ca^2+^ signals in astrocytes are evoked by the paired burst-stimuli (gray bars) in 8-to-12 Hz. **D)** The increment in pair-responses (R1 versus R2) from astrocytes is dominant when the paired stimuli in 8-to-12 Hz are given. **E)** The ratio of increment pattern to decrement patterns shows no significant difference between neuronal network and astrocytic network. Three asterisks indicate p < 0.001, N.S. means no significant difference, student’s t-test.

Figure 3 illustrates the three activity patterns of neurons and astrocytes in the barrel cortex. An example in 3A shows that most of the neurons express the increment in response to 8-to-12 Hz stimuli. The portions of decrement (R1 > R2), increment (R1 < R2) and parallel (R1 = R2) from all of the experiments (n = 6) are 21.5 ± 9.9% (blue bar in 3B), 61.9 ± 14.1% (red) and 16.6 ± 6.5% (green), i.e., the portion of increment pattern is higher than others (*p* < 0.001). In addition, the responses of the astrocytes to 8-to-12 Hz stimuli appear to be increment, decrement and parallel (Figure 3C). The portions in decrement, increment and parallel are 22.0 ± 7.6% (blue bar in Figure 3D), 63.4 ± 8.4% (red) and 14.6 ± 3.7% (green), respectively (*p* < 0.001, n = 6). Thus, a dominance of increment activity pattern of neurons and astrocytes is associated with an increasing frequency of whisker input.

We have calculated the ratio of increment portion to decrement one to estimate whether the responses of neurons and astrocytes to frequency-increased stimuli are parallel. As showed in Figure 3E, these ratios for neurons and astrocytes are not difference (*p* = 0.558). A parallel increase in the response strength of neurons and astrocytes indicates that the neurons and astrocytes in the barrel cortex coordinately respond to frequency messages in whisker tactile inputs.

### Barrel neurons and astrocytes synchronize their activities in response to high frequency input

**Figure 4:**
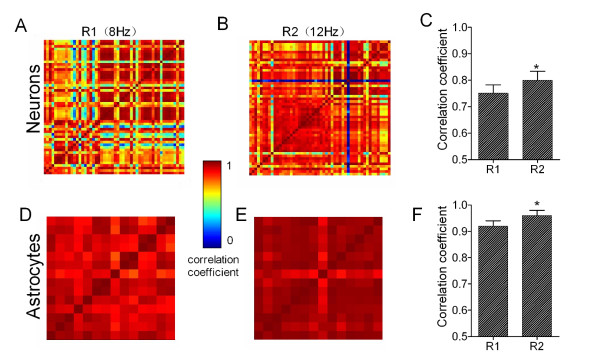
**The activity synchrony of neuronal and astrocytic networks increases in responses to whisker stimuli with high frequency. A ~ B)** Correlation matrices show cross-correlations for each of neuron-pairs in response (Ca^2+^ signals) to paired burst-stimuli, including response one (R1) vs. response two (R2) to 8 Hz (A) and 12 Hz (B) of stimulus frequency, respectively. **C)** shows a comparison of correlation coefficients averaged from all active neuron-pairs (excluding auto-correlations) in responses to paired burst-stimuli at 8-to-12 Hz. **D ~ E)** Correlation matrices show the cross-correlations for each of astrocyte-pairs in response to paired-stimuli, including R1 vs. R2 to 8 Hz (A) and 12 Hz (B) of stimulus frequencies, respectively. **F)** shows a comparison of the correlation coefficients averaged from all active astrocyte-pairs in responses to paired burst-stimuli (8-to-12 Hz). Asterisk presents *p* < 0.05 in paired student’s t-test.

To study whether the activity synchrony of barrel neurons and astrocytes was related to identify frequency message in whisker inputs, we analyzed pair-wise correlation among cell-pairs (Methods) in response to distinct frequencies. Each pixel in the matrices of Figure 4A ~ B and 4D ~ E represents peak cross-correlation for a pair of neurons, and dark-red pixels indicate the best cross-correlation (synchrony), or vice versa.

An example in Figure 4A ~ B shows cross-correlations among neuron-pairs in response to burst-pulse one (8 Hz) and two (12 Hz). Correlation coefficients in central peaks averaged from all of the experiments (n = 5) increase from 0.75 ± 0.04 for 8 Hz stimuli to 0.80 ± 0.03 for 12 Hz (*p* < 0.05, Figure 4C). Neuronal activity synchrony corresponding to high frequency stimuli indicates that barrel neurons discriminate input frequency changes by setting their synchrony.

Figure 4D ~ E shows an example that correlation coefficients among astrocyte-pairs increase in response to 8 Hz-to-12 Hz. The peak values of the correlogram for 8 Hz stimuli vs. 12 Hz ones are 0.92 ± 0.04 and 0.96 ± 0.03, respectively (*p* < 0.05, Figure 4F). The activity synchrony among the astrocytes in response to high frequency stimuli indicates that the barrel astrocytes modify their activity synchrony to encode the frequency message in whisker tactile inputs.

It is noteworthy that cells’ responses in barrel cortex to pair-stimuli in the same frequency (8-to-8 Hz) demonstrate decrement and less synchrony (Figures S1 ~ S2). The result does not support a possibility that the increment and synchrony in their responses to stimulus two at 12 Hz are due to repetitive stimulation. Moreover, the parameters used to quantify active strength and synchrony are not affected by the interval of two stimuli in barrel neurons ( Additional file 3: Figure S3).

### The neurons and astrocytes coordinately encode the frequency message in whisker tactile inputs

Barrel neurons and astrocytes are synchronously activated by whisker tactile inputs (Figure 2C). The spatial and temporal patterns of barrel neurons and astrocytes are similar in response to frequency signals in whisker stimulations (Figures 3~4). We further examined whether the neurons and astrocytes in the barrel cortex coordinately encoded the frequency signals.

**Figure 5:**
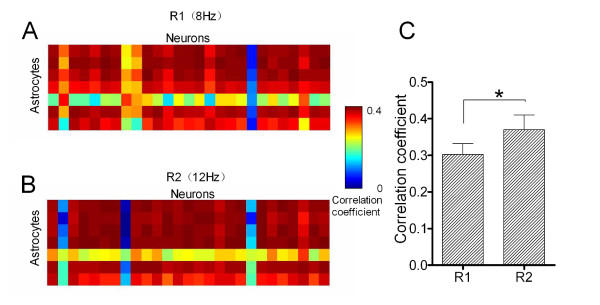
**The activity synchrony of barrel neuron-astrocyte pairs is associated with a frequency increase of whisker stimuli. A ~ B**) Correlation matrices show the cross-correlations for each of neuron-astrocyte pairs in response (Ca^2+^ signals) to paired-stimuli, including response one (R1) vs. response two (R2) at 8 Hz (A) and 12 Hz (B) of stimulus frequencies, respectively. Vertical axis in correlation matrix shows for astrocytes and horizonal axis for neurons. **C)** shows a comparison of correlation coefficients averaged from all active neuron-astrocyte pairs in responses to paired burst-stimuli at 8-to-12 Hz. Asterisk presents *p* < 0.05 in paired student’s t-test.

The cross-correlation for the pairs between neurons and astrocytes in response to whisker stimuli in 8-to-12 Hz was analyzed. Each pixel in Figure 5A ~ B represents peak cross-correlation for a pair of neuron-astrocytes, and the cross-correlations appear to the increases in response to burst-stimuli one (8 Hz) and two (12 Hz). Correlation coefficients in central peaks averaged from all of the experiments (n = 6) increase from 0.31 ± 0.03 for 8 Hz stimuli to 0.37 ± 0.04 for 12 Hz (*p* < 0.05, Figure 5C). The more synchrony between neurons and astrocytes in response to the higher frequency stimuli indicates that neurons and astrocytes in the barrel cortices coordinately encode frequency messages in whisker tactile inputs.

### The change of functional connections among nerve cells is involved in encoding input frequencies

Cellular mechanisms for barrel neurons and astrocytes coordinately to encode frequency messages in whisker tactile inputs is likely founded on the dynamical changes in their functional connections. If it is a case, the functional connections among nerve cells should be upregulated in response to an increase of stimulus frequency. The functional connections among these cells were estimated by using their cross-correlations [37,38]. Correlation coefficients during spontaneous activities at these nerve cells are set as no functional connections among them [39], and the averaged values above such coefficients plus two-time standard deviations are defined as the functional connections (Methods for details). Based on this principle, we analyzed the functional connections among neurons and astrocytes in the barrel cortex under the conditions of different input frequencies.

**Figure 6:**
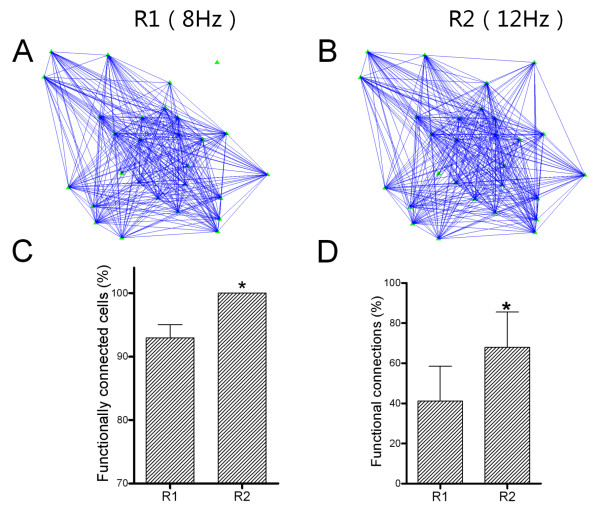
**Functional connections among barrel neurons increase in response to stimulus frequencies at 8 Hz and 12 Hz, in which response one (R1) corresponds to 8 Hz and response two (R2) corresponds to 12 Hz. A ~ B**) presents the functional connections (blue lines) among the barrel neurons (green symbols) under the conditions of stimulus frequencies at 8 Hz **(A)** and 12 Hz **(B)**, respectively. Scale bar is 20 μm. **C)** shows the percentages of functionally connected neurons in barrel networks during R1 (8 Hz input) and R2 (12 Hz). **D)** illustrates the percentage of functional connections for each neuron with others in R1 (8 Hz input) and R2 (12 Hz). Asterisk presents *p* < 0.05 in paied student’s t-test.

Figure 6 illustrates the functional connections among neurons in response to stimuli at 8 Hz-to-12 Hz, in which R1 corresponds to 8 Hz and R2 to 12 Hz. Figure 6A ~ B illustrates the functional connections (blue lines) among the neurons (green symbols) during R1 (A) and R2 (B). Figure 6C shows that the portions of the functionally connected neurons, in which 92 ± 2.1% of the activated neurons are connected with others in R1, compared to 100% in R2 (*p* < 0.05). Thus, more neurons in the barrel cortex are functionally connected in response to input frequency increase. Figure 6D shows the percentages of functional connections for each neuron with others in R1 and R2, which are 41.2 ± 17.3% and 67.9 ± 17.6%, respectively (*p* < 0.05). That is, each of neurons is functionally connected with more neurons in response to an increase of input frequency. In short, the neurons in the barrel cortex encode the messages of the increased input frequencies by strengthening their functional connections.

**Figure 7:**
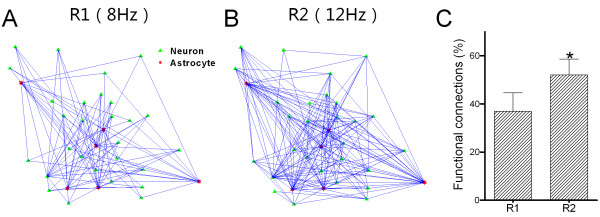
**Functional connections between barrel neurons and astrocytes increase in response to stimulus frequencies at 8 Hz and 12 Hz, in which response one (R1) corresponds to 8 Hz and response two (R2) corresponds to 12 Hz. A ~ B**) presents the percentages of functional connections (blue lines) between barrel neurons (green symbols) and astrocytes (red) under the conditions of stimulus frequencies at 8 Hz (A) and 12 Hz (B). Scale bar is 20 μm. **C)** illustrates the percentage of functional connections between barrel neurons and astrocytes in R1 (8 Hz input) and R2 (12 Hz). Asterisk presents *p* < 0.05 in paied student’s t-test.

Moreover, functional connections between neurons (green symbols) and astrocytes (reds) in response to stimuli at 8 Hz (Figure 7A) and 12 Hz (Figure 7B) were analyzed. The percentages of functional connections between neurons and astrocytes in R1 and R2 are 37.0 ± 7.6% and 52 ± 6.7%, respectively (Figure 7C, *p* < 0.05). Thus, the neurons and astrocytes in the barrel cortex coordinately encode an increase of input frequency via strengthening their functional connections.

## Discussion

With two-photon cellular imaging and LFP recording in the barrel cortex *in vivo*, we have studied the processes for network cells to encode frequency messages in whisker tactile inputs. In response to an increase of input frequency, the portions of neurons and astrocytes with increment activity patterns are dominant and the activities of neurons and astrocytes are synchronized (Figures 1~4), as well as the coordinate interactions between neuronal network and astrocytic one are strengthened (Figure 5). The process for these cells to encode an increase of input frequency is likely fulfilled by increasing their functional connections (Figures 6~7). Thus, the neurons and astrocytes in the barrel cortex coordinately respond to the frequency messages in whisker tactile inputs through changing the strength of their functional connections. Whether this process is present in other sensory cortices remains to be examined.

In terms of the neural encoding, many studies have been done at individual neurons and synapses. Digital spikes encoded at the neurons and analogue signals at the synapses are essential codes in the brain [40-44]. As the brain functions are presumably fulfilled by neuronal networks [1-3], the studies in the activity patterns of network neurons have been paid attention [13,16,18,45]. Their temporal and spatial activity patterns in response in a network to a single stimulus are known [14,17-19,21]. By giving stimuli in different frequencies, we reveal that nerve cells in the barrel cortex encode the frequency messages in whisker tactile inputs by resetting their functional connections as well as the portions of activity patterns and synchrony. Our findings provide the clues for understanding how neurons and astrocytes in the network coordinately program the brain codes for well-organized behaviors.

In the sensory systems, sequential stimuli in the same feature induce an adaptation in the sensations, and the increases in intensity or frequency should be given to elevate the sensitivity to these stimuli [46-48]. The changes in the ability of firing digital spikes at individual neurons are associated with the adaptation and sensitization [23,49,50]. It is not clear how the neurons and astrocytes integrated into neural networks encode these processes. Our studies demonstrate that pair-stimuli in the same frequency to whiskers make neurons and astrocytes to be lowered and asynchrony in their activities (Figures S1 ~ S2), and that an increasing stimulus frequency causes their responses to be increased and synchronized (Figures 2~4). The increment versus decrement and synchrony vs. asynchrony of nerve cells in response to sequential stimulations may be basic forms for encoding frequency signals and for the adaptation vs. sensitization. Their conversions may be involved in reading out the detail messages in natural sensory inputs.

The astrocytes presumably provide a micro-environment for the neurons to be functional [10,35,36,51]. Studies by two-photon cellular imaging show that sensory stimuli induce a change of Ca^2+^ signal in astrocytes [8] and that Ca^2+^ dynamics in the neurons of barrel cortex are synchrony [4,7]. The astrocytes play a crucial role in coupling neuronal organization to map signals in visual cortex [6]. Beyond these findings, we show that spatial and temporal activities among astrocytes are coupling with neuronal activities in strength and synchrony to encode frequency messages in whisker tactile inputs, which is based on the dynamical change of their functional connections.

It is noteworthy that the responses of barrel neurons and astrocytes may be thought to be different in the anesthetized animals versus awaked ones [52,53]. In our hypothesis, all responses of brain cells to stimulus intensity and frequency should be reduced in parallel from the anesthetized animals, compared to the awaked animals, such that the patterns of responses (i.e., response one versus response two) may not be changed. This hypothesis will be examined in our future studies.

## Conclusions

Corresponding to an increase of whisker stimulus frequency, the neurons and astrocytes in the barrel cortex upregulate their activity strength, synchronize their activities and strengthen their functional connections. Therefore, the coordinate interactions among neurons and astrocytes in cortical networks are mechanistically involved in encoding the frequency messages in sensory inputs.

## Methods

### Animal surgery and fluorescence labeling

The study and all experiments conducted were fully approved by the Institutional Animal Care Unit Committee (IACUC) in Administration Office of Laboratory Animals Beijing China (ID# B10831). FVB mice in postnatal days (PND) 20 ~ 35 were anesthetized by the intraperitoneal injections of urethane (1.5 g/kg). Anesthetic depth was judged based on lack of reflexes in pinch withdrawal and blink eyelid, and was maintained by giving the supplemental dosage of urethane (0.25 g/kg) throughout the experiments. Body temperature was maintained by using a computer-controlled heating blanket at 37°C. The barrel cortex was located based on the distributional map of superficial vessels and confirmed by histological reconstructions [54](Figure 1A). A craniotomy (1 ~ 2 mm in diameter) was made on the skull above the center of the barrel cortex (Figure 1B), which was located at 1 mm posterior to the bregma and 3.5 mm lateral to the midline. It is noteworthy that the dura was intact throughout all experiments, and the care was taken to avoid any damage to superficial vessels and cortices.

In our studies, Oregon Green BAPTA-1-AM (OBG-1, Invitrogen, CA USA), a Ca^2+^ dye, was used to measure the activities from neurons and astrocytes. OGB-1 was dissolved in DMSO and 20% Pluronic F-127 (2 g Pluronic F-127 in 10 ml DMSO, Invitrogen, USA) to have its stock solution at 10 mM. The stock solution was diluted in ACSF to yield its final concentration at 1 mM. This OBG-1 solution was injected into layer I ~ II of barrel cortex by the pressure (1 bar, 5 min) through a patch pipette (200 μm below the pia) to label the population of nerve cells, termed as multicell bolus loading [55-57]. In the meantime, 100 μM sulforhodanmine-101 (SR101, Invitrogen, USA) was co-injected to label the astrocytes specifically [33]. The volumes of these dyes were controlled at ~0.5 μl. After micro-injections, the craniotomy well was filled by low-melted agarose (1 ~ 2%) in saline and then was sealed with glass coverslip. The exposed skull was adhered to a custom-made recording chamber with dental acrylic cement, which was surperfused with saline (mM): 125 NaCl, 2.5 KCl, 26 NaHCO_3_, 1.25 NaH_2_PO_4_, 2 CaCl_2_, 1 MgCl_2_, 20 glucose (pH 7.4). The saline was warmed up to 37°C and bubbled with 95%O_2_/5% CO_2_.

### Two-photon cellular imaging

Calcium imaging experiments were done 1 hour after dye injection by using two-photon laser scanning confocal microscope (Olympus FV-1000, Tokyo, Japan). Two-photon laser-beam generator (Mai Tai, Physical Spectrum, USA) and scanning system were mounted onto an upright microscope (Olympus BX61WI) with water immersion objectives (IR-LUMPLan Fl, 0.8NA, 40X). Two-photon laser beam at 810 nm was given to excite OGB-1 and SR101. Average power delivered to the brain tissue was less than 75 mW. The emission wavelengths were 523 nm for Ca^2+^-binding OGB-1 and 603 nm for SR-101, respectively. Whole field images were acquired at 10 Hz frame rate (256 × 256 pixels). The parameters for laser beam and photomultiplier tube were locked for the measurements before and after different stimuli as well as throughout all experiments in order to have a consistent condition in the comparisons of the results.

### Local field potential (LFP) recording

Local field potentials were recorded in layerII/III of barrel cortex by using a glass pipette that contained the standard pipette solution (150 mM NaCl, 2.5 mM KCl, 10 mM HEPES). The resistance of recording pipettes was ~10 MΩ. Electrical signals were inputted to an AxoClamp-2B amplifier and pClamp 10 system (Axon Instrument Inc. CA USA), in which Clampex was used for data acquisition and Clampfit for data analysis. The electrical signals were digitized at 10 kHz and band-pass filtered at 1 ~ 100 Hz. It is noteworthy that the LFP recording and two-photon cellular imaging were performed in the identical area of barrel cortex (Fig. 1A ~ B), which allowed us to study the processes of neural encoding in the barrel cortex by electrophysiology and cellular imaging.

### Whisker stimuli and barrel cells’ responses

All major whiskers in the contralateral sides of the imaged barrel cortices were deflected in a caudal-to-rostral direction by air-puffing during the experiments, which is more similar to the natural movement of whiskers. Whisker stimuli were done by giving the sequential brief pulses of air-puffing (50 psi, 50 ms) through a tiny steel tube that was mounted on a micromanipulator and controlled with costume-made LabVIEW program. The stimulus patterns were the paired burst-pulses, in which each burst had a given frequency. The frequency patterns in these paired bursts (10 seconds for each) were 8 and 12 Hz (8-to-12 Hz), closely to natural frequency in exploratory whisking [25,49,58]. Burst-pulse intervals were 10 seconds. To avoid the stimulations to the skin and furs, a tip of the stimulator was positioned in a way that it did not blow on the snout.

The amplitudes of Ca^2+^ signals are correlated positively with spike frequency, and Ca^2+^ levels in a neuron indicate its functional activities, so that spiking activity at the neurons can be estimated from their somatic Ca^2+^ signals *in vivo*[19,31,32,59]. Different from the spikes as a functional index of neuronal activity, the activities of astrocytes are associated with the changes of Ca^2+^ signals [30,60]. In addition, the synchrony of Ca^2+^ signals provide a measurement for the timing of cellular activities [4,7,54,61]. Therefore, we studied the activity patterns of barrel neurons and astrocytes in response to whisker stimuli by using *in vivo* two-photon cellular imaging in order to reveal the processes of neuronal encoding in brain networks, in which the peak amplitudes and temporal synchrony of Ca^2+^ signals were analyzed.

### Data analyses

Ca^2+^ fluorescence signals for cellular responses to whisker stimuli were acquired by using Fluoviewer-10 software (Olympus Inc. Japan) and analyzed in the regions of interest (ROI) from cell bodies by using NIH ImageJ and MATLAB (MathWorks). To reduce the photon and PMT noise, a median filter (radius, 1 pixel) was applied to all images. Ca^2+^ signals in cellular responses were normalized and presented as relative fluorescence changes (ΔF/F)[29,62,63]. Baseline fluorescence (F) was an averaged value in the ROI before stimuli, and ΔF values were differences between Ca^2+^ signals from the evoked responses in the ROI and baseline fluorescence. It is noteworthy that all of the fluorescence signals were subtracted from the noise signals of unstained blood vessels, as well as Ca^2+^ signals in the astrocytes were normalized to SR101 signal to eliminate motion artefacts. The normalized Ca^2+^ signals were smoothed by low-pass Butterworth filter to remove low-amplitude fluctuations and to minimize the distortions from fast Ca^2+^ transients [31,32]. Effective signals from each of active cells were judged according to a criterion that their relative fluorescence changes were greater than 2.5 SD of baseline values and lasted for 500 ms. In our experiments, whisker stimuli induced the robust changes of Ca^2+^ signals in barrel cells, and the criteria above was found effective for sorting evoked signals from noise.

In our study, the paired burst-stimuli were given to induce two sequential responses. As the fluctuation of fluorescence signals influenced a precise measurement of response amplitudes, we thought the magnitude differences of two responses if their differences were above 2 SD of baseline values; however, we defined no difference in the magnitude of two responses if their net changes were less than 2 SD. If cellular response one (R1) was larger than response two (R2) above 2 SD of baseline, the pattern was defined as the decrement (R1 > R2). On the other hand, R2 > R1 above 2 SD was the increment. No differences for R1 and R2 were called as parallel. This classification is similar to synaptic transmission patterns [43]. It is noteworthy that R2 values were the absolute changes of responses induced by stimulus two. That is, if the calcium signals in response to stimulus one were not back to their baseline levels, R2 was measured as a difference between the magnitude of response two and the residual level of response one.

The pairswise cross-correlations of normalized and smoothed Ca^2+^ signals (ΔF/F) in the neurons and/or astrocytes between each of cell pairs were analyzed as Pearson correlation [7,54,61,64]. Although the cross-correlations between neurons from raw fluorescence traces were higher than the deconvolved traces over 2 fold [61], we computed raw traces without temporal deconvolution in the neurons consistently with those in astrocytes which had no spikes firing [65,66]. Consider two signals x (t) and y (t) of a real variable t; the cross-correlation *r* at delay *d* is defined as:

(1) $$r=\frac{\sum_{t}[(x(t)-mx)\times (y(t-d)-my)]}{\sqrt{\sum_{t}{\left(x(t)-mx\right)}^{2}}\times \sqrt{\sum_{t}{\left(y(t-d)-my\right)}^{2}}}$$

*mx* and *my* are the means of the corresponding series. Correlation coefficients normalized to the autocorrelation at zero lag were calculated. Based on these calculations, the correlation matrices were plotted using MATLAB 7.0.

In the study of functional connectivity [38,67] among network cells, we converted correlation coefficient matrix (*r*) into binary adjacency matrix (*A*) by setting a threshold (*thresh*) [68,69]. It was the averaged correlation coefficients plus two-time standard deviations corresponding to spontaneous cellular activities without whisker stimuli. If *r*_*ij*_ during whisker stimuli is lareger than *thresh*, i.e., *A*_*i,j*_ equals to one, the functional connection is present between cell *i* and cell *j*. On the other hand, if *r*_*ij*_ during whisker stimuli is less than *thresh*, i.e., *A*_*i,j*_ is equal to zero, the functional connection is not present between cell *i* and cell *j*. The formula is

(2) $$A_{i,j}=\{\begin{array}{c} 1\;\text{,}\;r_{\mathrm{ij}}>thresh\\ 0\;\text{,}\;otherwise\text{.} \end{array}$$

It is noteworthy that the definition of functional connection, whose threshold is set at mean + 2SD of correlation coefficients during the spontaneous activities of network cells, is based on an assumption that their activities are random in nature (no coordination). In other words, there are no interactions, or functional connections, among these network cells without input signals [70].

Based on these criteria of binary adjacency matrices and the spatial positions of network cells, we plotted the graphs which consisted of a set of nodes (the cells activated by stimulus bursts) and their functional connections (lines) under the conditions of response one to 8 Hz stimuli and response two to 12 Hz. In these graphs of neural networks, two parameters for each cell were merited to indicate how each cell is connected with others. The cell that connects with one at least is called as a function-connected cell in neural network. The percentages of function-connceted cells present how many cells are functionally connected with others. If a cell connects with others, the percentages of functional connections for its actually connected cells in the total cells are calculated to present the connection strength for each of network cells. The folmula are given below.

For a neural network consisting of activated neurons (*N)* in complete graphs, the number of connctions for each neuron with others is *N-1*. The number of function-connected neurons is *n,* and the averaged number of connections for each neuron with others is *k*. Thus, *P*_*n*_ *= n/N* stands for the percentages of function-connected neurons. *P*_*k*_ *= k/(N-1)* presents the percentages of functional connections of each neuron. The astrocytes are connected tightly and widely via gap junctions, the number of connected neuron-astrocyte pairwise shows no variation during different stimuli in our studies. In a network comprising of *N* neurons and *M* astrocytes, we calculate the percentages of neuron-astrocytic functional connections (*l*) in total potential links, which is *P*_*l*_ *= l/(N∙M)*.

All data are presented as mean ± SEM. Student’s t tests (two-tailed, paired, or unpaired assuming unequal variances) were done in R software package, version 2.10.1 (http://www.r-project.org/) to evaluate statistical significance. A *p* ≤ 0.05 is defined as statistical significance.

## Competing interests

The authors declare that they have no competing interests.

## Authors’ contributions

JZ and JHW conceived the idea, designed experiments, and wrote the paper. JZ and DW executed experiments and analyzed data. All authors read and approved the manuscript.

## Supplementary Material

Additional file 1 **Figure S1.** Barrel network neurons show a dominant decrement in responses to the same frequency of paired whisker stimuli. **A)** shows two sequential Ca^2+^ signals at barrel neurons (a trace per cell) in response to paired burst-stimili at 8-to-8 Hz. **B)** The paired burst-stimuli induce increment ( n =2/24; blue symbols in top panel), decrement (n=18/24; reds in middle) and parallel (n= 4/24; greens in bottom). ### shows p<0.001 in two-tail paired t-test. Click here for file

Additional file 2 **Figure S2.** The activity synchrony of barrel neurons decreases in correspondent to whisker stimuli with the same frequency. **A~B)** Correlation matrices show the correlation coefficients for each of neuron-pairs in response (i.e., Ca^2+^ signals) to paired-stimuli, including response one (R1) vs. response two (R2) to stimuli both at 8 Hz . **C)** shows the comparison of mean cross-correlation coeffiecent for all active neuron-pairs (excluding autocorrelations) in responses R1 and R2 to paired burst-stimuli at 8-to-8 Hz. Click here for file

Additional file 3 **Figure S3.** The interval of the whisker stimuli does not affect the measurement of activity patterns and synchrony. **A-B)** shows the averaged responses in a barrel neuronal network induced by the 8-to-12 Hz whisker stimuli with different intervals. The stimuli interval of the panel **A** is 10 sec and **B** is 60 sec. **C)** Both the short interval (10sec) and long interval (60sec) of the 8-to-12Hz stimuli induces a dominant decrement in barrel neurons (p<0.001, student’s test). **D)** shows the comparision of mean cross-correlation coeffiecent for all active neuron-pairs in responses R1, R2 (responses induced by the second stimuli after a short interval ) and R2’ (responses induced by the second stimuli after a long interval ). The coeffiecent of R1 and R2 are significantly higher than the one of R1 (P<0.05, student’s test). The coffeicents of R2 and R2’ shows non significant difference. Click here for file
