# Supplementary figures and images for: Barrel cortical neurons and astrocytes coordinately respond to an increased whisker stimulus frequency
Source: Mol Brain. 2012 Apr 26;5:12. doi: 10.1186/1756-6606-5-12 (PMC3465214; doi:10.1186/1756-6606-5-12)

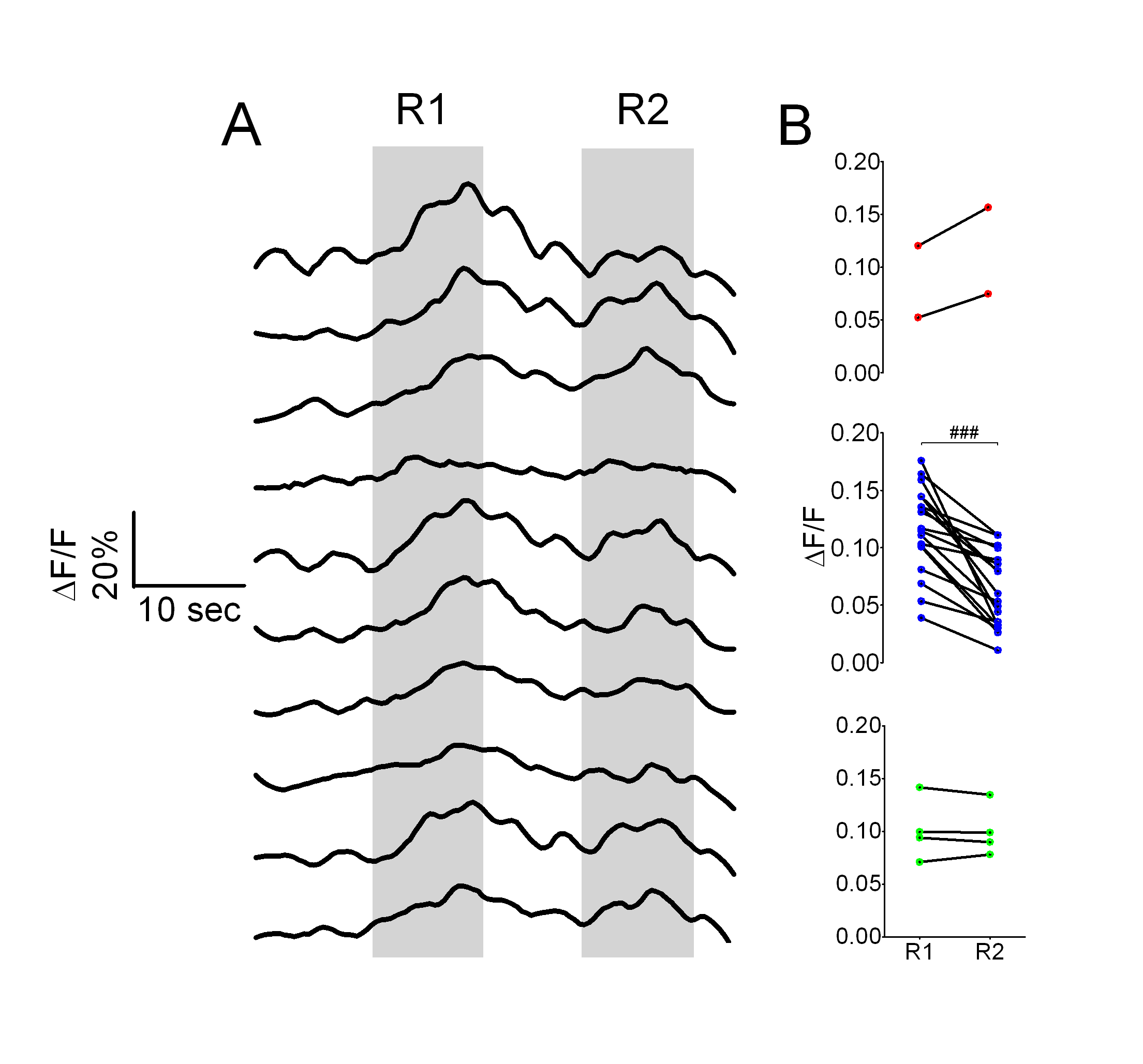

Supplement: Additional file 1 — Figure S1. Barrel network neurons show a dominant decrement in responses to the same frequency of paired whisker stimuli. A) shows two sequential Ca2+ signals at barrel neurons (a trace per cell) in response to paired burst-stimili at 8-to-8 Hz. B) The paired burst-stimuli induce increment ( n =2/24; blue symbols in top panel), decrement (n=18/24; reds in middle) and parallel (n= 4/24; greens in bottom). ### shows p<0.001 in two-tail paired t-test. [file 1756-6606-5-12-S1.tiff]

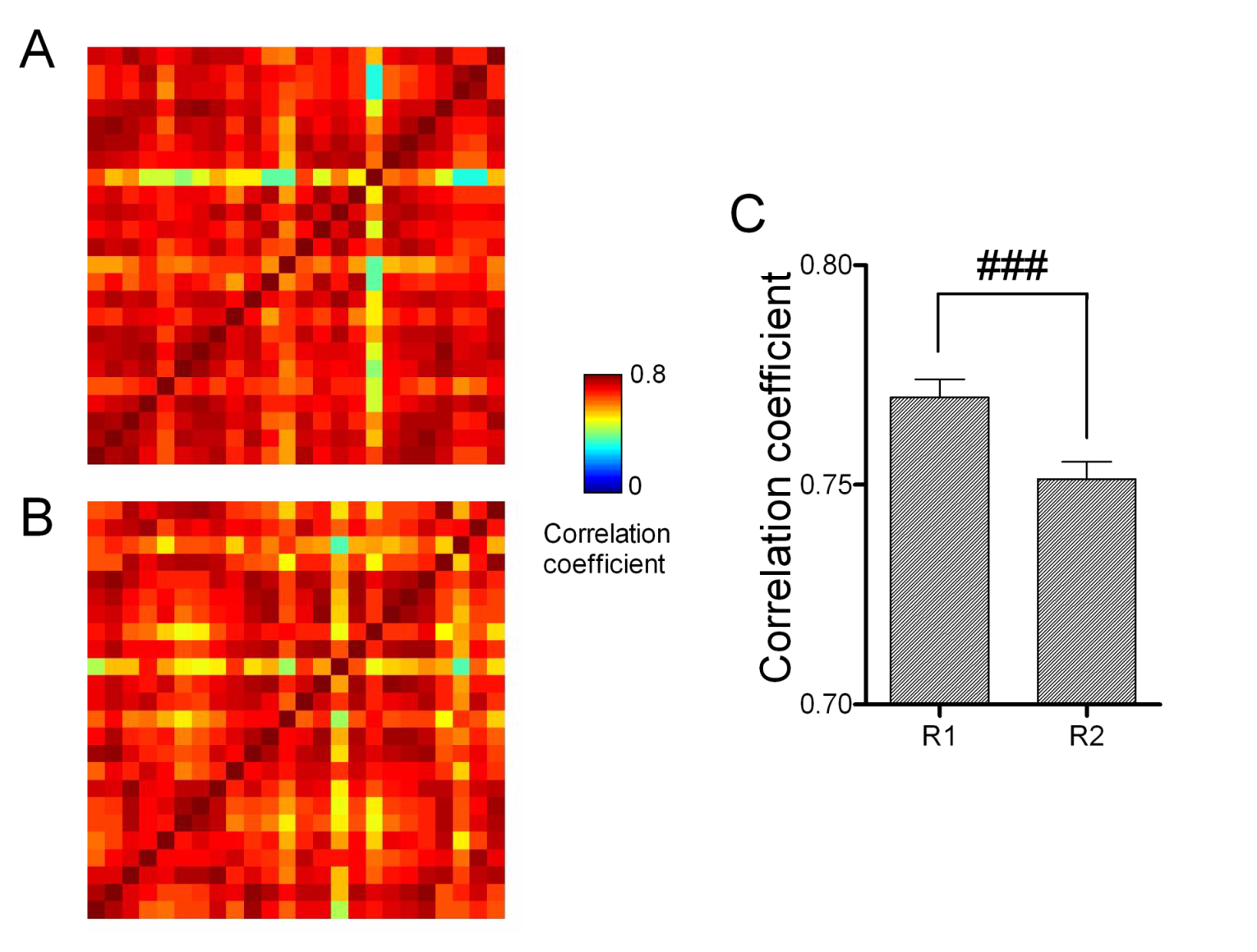

Supplement: Additional file 2 — Figure S2. The activity synchrony of barrel neurons decreases in correspondent to whisker stimuli with the same frequency. A~B) Correlation matrices show the correlation coefficients for each of neuron-pairs in response (i.e., Ca2+ signals) to paired-stimuli, including response one (R1) vs. response two (R2) to stimuli both at 8 Hz . C) shows the comparison of mean cross-correlation coeffiecent for all active neuron-pairs (excluding autocorrelations) in responses R1 and R2 to paired burst-stimuli at 8-to-8 Hz. [file 1756-6606-5-12-S2.tiff]

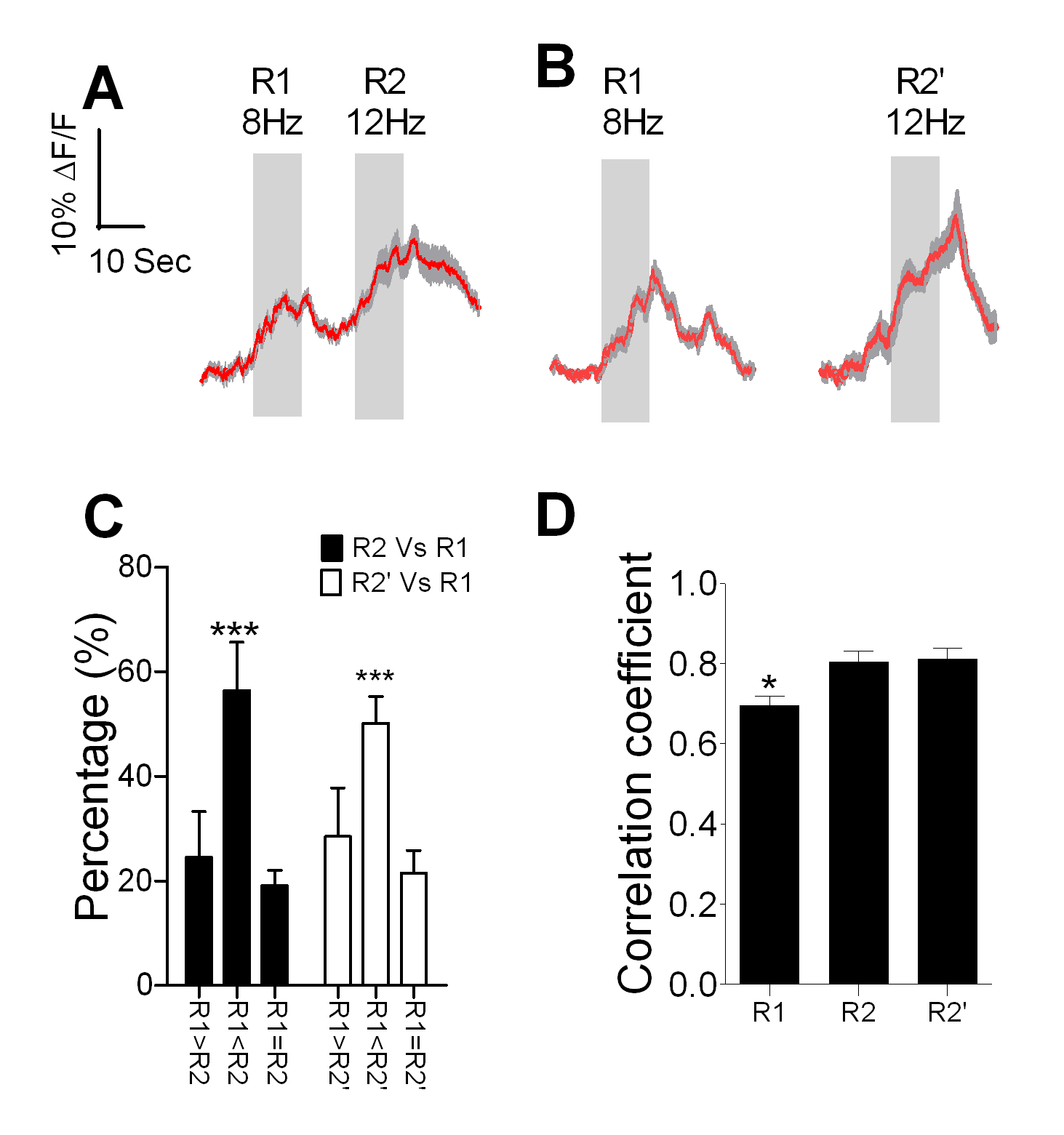

Supplement: Additional file 3 — Figure S3. The interval of the whisker stimuli does not affect the measurement of activity patterns and synchrony. A-B) shows the averaged responses in a barrel neuronal network induced by the 8-to-12 Hz whisker stimuli with different intervals. The stimuli interval of the panel A is 10 sec and B is 60 sec. C) Both the short interval (10sec) and long interval (60sec) of the 8-to-12Hz stimuli induces a dominant decrement in barrel neurons (p<0.001, student’s test). D) shows the comparision of mean cross-correlation coeffiecent for all active neuron-pairs in responses R1, R2 (responses induced by the second stimuli after a short interval ) and R2’ (responses induced by the second stimuli after a long interval ). The coeffiecent of R1 and R2 are significantly higher than the one of R1 (P<0.05, student’s test). The coffeicents of R2 and R2’ shows non significant difference. [file 1756-6606-5-12-S3.tiff]
